# Predicting Nitrogen‐Based Families of Compounds: Transition‐Metal Guanidinates *T*CN_3_ (*T*=V, Nb, Ta) and Ortho‐Nitrido Carbonates *T′*_2_CN_4_ (*T′*=Ti, Zr, Hf)

**DOI:** 10.1002/anie.202011196

**Published:** 2020-10-29

**Authors:** Dongbao Luo, Xianji Qiao, Richard Dronskowski

**Affiliations:** ^1^ Hoffmann Institute of Advanced Materials Shenzhen Polytechnic 7098 Liuxian Blvd, Nanshan District Shenzhen China; ^2^ Chair of Solid-State and Quantum Chemistry Institute of Inorganic Chemistry RWTH Aachen University 52056 Aachen Germany

**Keywords:** carbodiimide, guanidinate, non-linear optics, ortho-nitrido carbonate, water splitting

## Abstract

Due to its unsurpassed capability to engage in various sp hybridizations or orbital mixings, carbon may contribute in expanding solid‐state nitrogen chemistry by allowing for different complex anions, such as the known NCN^2−^ carbodiimide unit, the so far unknown CN_3_^5−^ guanidinate anion, and the likewise unknown CN_4_^8−^ ortho‐nitrido carbonate (*onc*) entity. Because the latter two complex anions have never been observed before, we have chemically designed them using first‐principles structural searches, and we here predict the first hydrogen‐free guanidinates *T*CN_3_ (*T*=V, Nb, Ta) and ortho‐nitrido carbonates *T′*_2_CN_4_ (*T′*=Ti, Zr, Hf) being mechanically stable at normal pressure; the latter should coexist as solid solutions with the stoichiometrically identical nitride carbodiimides and nitride guanidinates. We also suggest favorable exothermic reactions as useful signposts for eventual synthesis, and we trust that the decay of the novel compounds is unlikely due to presumably large kinetic activation barriers (C−N bond breaking) and quite substantial Madelung energies stabilizing the highly charged complex anions. While chemical‐bonding analysis reveals the novel CN_4_^8−^ to be more covalent compared to NCN^2−^ and CN_3_^5−^ within related compounds, further electronic‐structure data of *onc* phases hint at their physicochemical potential in terms of photoelectrochemical water splitting and nonlinear optics.

## Graphical abstract

Structural searches from first principles have led to the prediction of solid‐state carbodiimides, guanidinates, and ortho‐nitrido carbonates, the latter introducing the diamond motif into condensed‐matter nitrogen chemistry by high pressure. Some of these novel phases are expected to show potential in terms of photoelectrochemical water splitting and nonlinear optics.

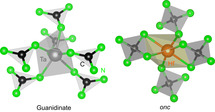

## Introduction

**Figure 1:**
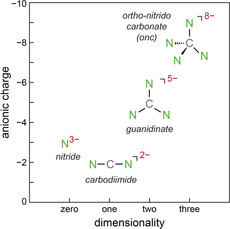
Sketch of charge and dimensionality of four nitrogen‐based (complex) anions as a function of their shape.

The search for new solid‐state nitrides or nitrogen‐based materials in general remains intense due to the wide range of exciting and quite diverse applications, such as N‐based fertilizers, explosives, high‐performance steel coatings, superconductors, electrides, UV‐LED materials, and a lot more.^[1]^ Despite enormous promise in their chemical and physical functionalities, the sheer amount of N‐based materials is much smaller than those of the oxides, for reasons well known to chemists.^[2]^ Somewhat simplified, the most fundamental N‐based anions which we dub “first generation” only contain nitrogen coordinated to a metal atom, such as simple nitrides,^[3]^ pernitrides,^[4]^ azides,^[5]^ diazenides,^[6]^ and the recently predicted LiN_5_.^[7]^ The socalled “second generation” may then incorporate an additional nonmetal atom such as, for instance, in nitrido borates^[8]^ or oxido nitrides.^[9]^ Another prominent branch of the second generation is based, at least in principle, on C‐centered complex anions by utilizing the carbon atom's diverse hybridization (sp, sp^2^, sp^3^), thereby, for example, forming the linear carbodiimide or cyanamide NCN^2−^ anion, see Figure 1. By doing so, the anionic dimensionality grows from zero (N^3−^) to one (NCN^2−^).

Clearly, N^3−^ nitride and also NCN^2−^ carbodiimide chemistries are well developed. Not only is GaN the fundamental material for blue light‐emitting diodes (LED), its chemistry started in the 1930s already^[10]^ and was then extended, by Juza and successors, to practically all kinds of metals, growing ever since, now also covering metastable compounds such as Sn_3_N_4_ and Na_3_N.^[11]^ As for carbodiimides, the earliest phase CaNCN served as a fertilizer in the 19th century and was structurally confirmed in 1962.^[12]^ This field started to grow since the 1990s with better routes for main‐group metal carbodiimides.^[13]^ After the turn of the millenium, transition‐metal carbodiimides also became accessible,^[14]^ thereby opening up applications in (photo)electrochemistry.^[15]^

The next topological step consists of the planar (hence, two‐dimensional, see Figure 1) CN_3_^5−^ guanidinate anion, and a similar class of guanidinate phases was firmly established through making RbCN_3_H_4_ in 2011^[16]^ whose complex anion, descendant of the astonishingly basic guanidine molecule, still carries four N‐bonded H atoms. Syntheses in liquid ammonia further led to singly deprotonated (Li, Na, K, Rb, Cs, Ba, Eu)^[17]^ and also doubly deprotonated (Sr, Ca, Yb) guanidinates^[18]^ but a hydrogen‐free CN_3_^5−^ guanidinate unit has not been accomplished up to the present day. And yet, there should be the tetrahedral CN_4_^8−^ ortho‐nitrido carbonate (*onc*), a three‐dimensional complex anion (see Figure 1) which was never observed before, reminding us of the carbon atom's sp^3^ hybridization alluding to the diamond structure and a multitude of hydrocarbons or other CX_4_ species, thereby also highlighting the huge challenge to find such “tetrahedral” C‐based solid‐state compounds.^[19]^

To path the way to their discoveries, we have performed an extensive structure search based on structural evolution through the Particle Swarm Optimization (PSO) algorithm.^[20]^ Assuming that the terminal structures and compositions have been firmly determined, the corresponding synthetic routes can then be adjusted by chemical creativity. For the density‐functional calculations, 3d‐5d transition metals of the vanadium group (V, Nb, Ta) were selected to satisfy charge balance given a fixed stoichiometry of *T*CN_3_. Likewise, the corresponding transition metals of the titanium group (Ti, Zr, Hf) were taken using a fixed *T′*_2_CN_4_ formula. Fortunately, we found twelve structurally and chemically related compounds which turned out as dynamically stable at atmospheric pressure, surprisingly enough. Among those, the first pure guanidinates *T*CN_3_ (*T*=V, Nb, Ta) were confirmed to contain the CN_3_^5−^ anion, and the first ortho‐nitrido carbonates *T′*_2_(CN_4_) (*T′*=Ti, Zr, Hf) were also predicted to incorporate CN_4_^8−^. In addition, we found, somewhat unexpectedly, stable nitride carbodiimides of the form *T′*_2_N_2_(NCN) and nitride guanidinates *T′*_2_N(CN_3_) as predicted solid mixtures between N^3−^ and NCN^2−^ or CN_3_^5−^, respectively, emphasizing the idea of solid‐state chemical equilibrium between those anions. All compounds are predicted as being semiconductors, and some of them should provide potential in the fields of electrochemical water splitting and nonlinear optics.

## Results and Discussion

### Stability

The PSO structure predictions were performed by running CALYPSO^[21]^ based on density‐functional theory for unit cells containing up to four formula units *T*CN_3_ and *T′*_2_CN_4_, employing VASP together with projector augmented waves (PAW),^[22]^ the generalized‐gradient approximation (GGA),^[23]^ and the Monkhorst‐Pack scheme.^[24]^ Twelve low‐energy compounds were studied in detail whose well optimized structural parameters are shown in Table S1. Their stability was confirmed by three criteria, namely, phonon band structure by finite displacements (Phonopy),^[25]^ elastic constants, and synthetic route. That is to say that, first, all twelve phases are dynamically stable with no imaginary modes in the phonon bands (Figure S1), even at zero pressure. Second, as regards mechanical stability, the calculated elastic constants all satisfy the corresponding Born elastic stability criteria^[26]^ as shown in Table S2. With respect to chemistry, the selected metathetic pathway for the composition *T*CN_3_ was chosen as [Eq. 1]

(1) $$T\mathrm{Cl}{}_{5}+\mathrm{Na}{}_{2}\mathrm{NCN}+\mathrm{Na}{}_{3}N\to T\mathrm{CN}{}_{3}+5\;\mathrm{NaCl},$$

assuming convenient (i.e., high‐energy Na_3_N) starting materials. For the *T′*_2_CN_4_ composition, the metathetic route was targeted as [Eq. 2]

(2) $$2\;T{}^{'}\mathrm{Cl}{}_{4}+\mathrm{Na}{}_{2}\mathrm{NCN}+2\;\mathrm{Na}{}_{3}N\to T{}^{'}{}_{2}\mathrm{CN}{}_{4}+8\;\mathrm{NaCl}$$

**Figure 2:**
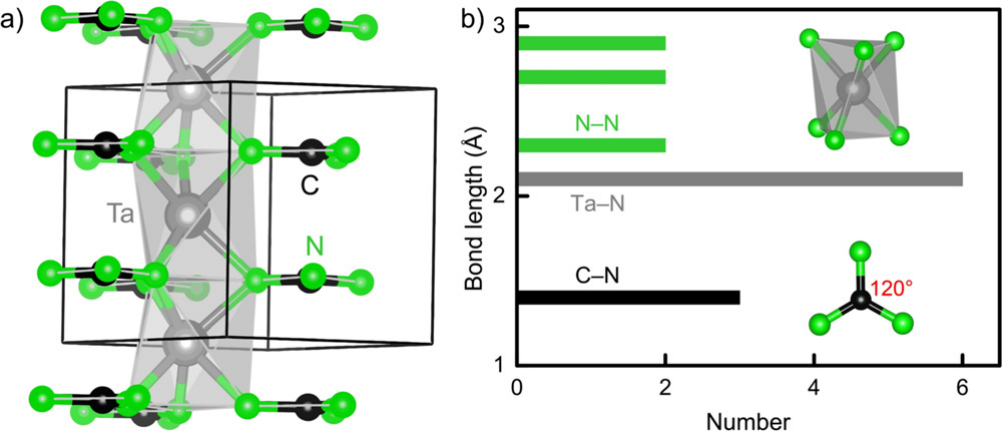
a) Crystal structure of *T*CN_3_ guanidinates (*T*=V, Nb, Ta) with space group *P* $\bar{6}$ *c*2 and b) bond‐length histogram of various interactions.

**Figure 3:**
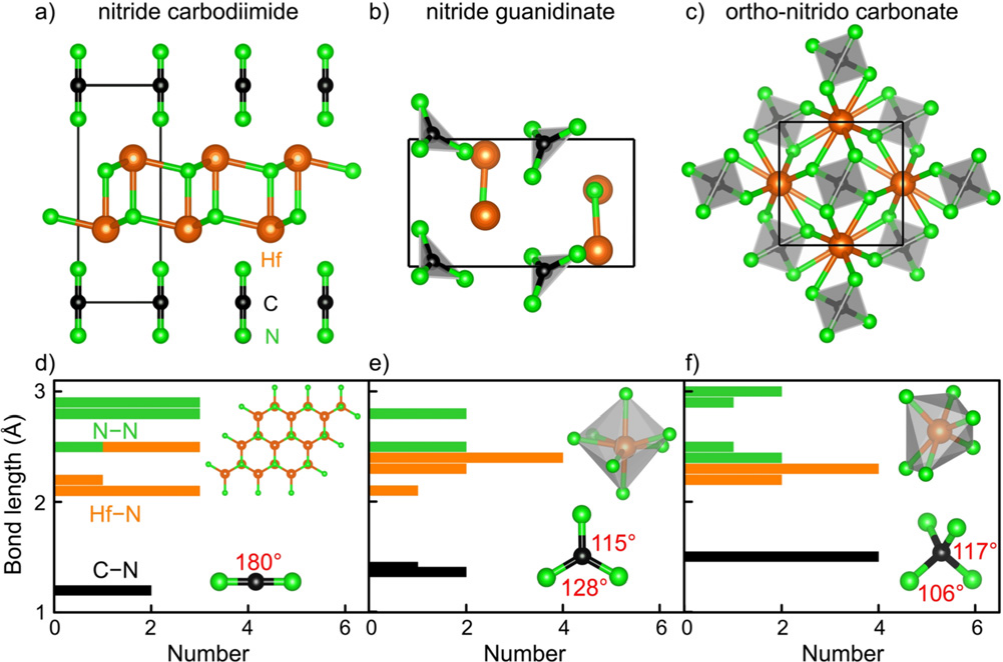
The crystal structures of the a) nitride carbodiimides, b) nitride guanidinates, and c) ortho‐nitrido carbonates of the *T′*_2_CN_4_ (*T′*=Ti, Zr, Hf) compositions using the Hf_2_CN_4_ example. The corresponding bond‐length histograms are given in d), e), and f).

Fortunately, the negative formation energies shown in Figure S2 indicate that all *T*CN_3_ and *T′*_2_CN_4_ are exothermic phases, so successful synthesis should be tried. Other routes, better still, might be possible as well. Structurally, all *T*CN_3_ are predicted to crystallize in the hexagonal system with space group *P* $\bar{6}$ *c*2 (see Figure 2) whereas, for *T′*_2_CN_4_, three different types of compounds should be observable, trigonal *P* $\bar{3}$ *m*1, tetragonal *P* $\bar{4}$ 2_1_ *c*, and orthorhombic *Cmc*2_1_ (see Figure 3), all three differing in and indicative of their chemical characters.

### Structural Details

Starting with the *T*CN_3_ types crystallizing in *P* $\bar{6}$ *c*2, the compounds VCN_3_, NbCN_3_, and TaCN_3_ adopt the same structure as shown in Figure 2 a), and we select the heavy TaCN_3_ for a detailed internal description, see Figure 2 b). First, and in perfect harmony with the sum of Shannon's ionic radii for Ta^5+^ and N^3−^,^[27]^ there are six equidistant Ta−N bonds of 2.10 Å inside a *trans* face‐sharing TaN_6_ octahedron stacked along *c*, a bit similar to the wider SrN_6_ octahedron (Sr−N=2.67 Å) in SrC(NH)_3_ but more twisted.^[18]^ As for the important CN_3_ core with *D*_3*h*_ symmetry, there are no H atoms as in SrC(NH)_3_ while C−N=1.35 Å and N−C−N=120° are practically identical.^[18]^ The shortest N−N distance of 2.35 Å is nonbonding, and Ta−Ta=2.8 Å is also far beyond the sum of the effective ionic radii for Ta^5+^ (0.64 Å), indicating essentially no N−N and Ta−Ta interactions. That being said, this first predicted transition‐metal guanidinate without H atoms appears as showing a “layered” motif with guanidinate anions and metal cations alternately stacked on top of each other, Figure 2 a).

As for the *T′*_2_CN_4_ formula, there are three different chemical motifs, corresponding to three different compound classes, and they are shown in Figures 3 a) to 3 c). For reasons of convenience, we select *T′*=Hf to study their chemical and structural peculiarities, as depicted in Figures 3 d) to 3 f). Similar to the previous discussion of *T*CN_3_, the shortest N−N distance of 2.32 Å and Hf−Hf distance of 2.97 Å are far beyond any significant interaction and will not be discussed any further. In contrast, the Hf−N and C−N bond lengths and connectivities help to separate the three different types of *T′*_2_CN_4_:

Figure 3 a) depicts the first *P* $\bar{3}$ *m*1‐type *T′*_2_CN_4_ representative which is predicted to crystallize with two spatially separated Hf−N and N−C−N layers, a nitride carbodiimide *T′*_2_N_2_(NCN). There are three shortest Hf−N=2.09 Å bonds in a plane around each Hf^4+^ by nearest N^3−^ neighbors to generate a heterographene‐like Hf−N layer, and two such layers form a double layer. For comparison, the Hf−N distances in Hf(NCN)_2_ lie between 2.03 and 2.24 Å.^[28]^ The second shortest Hf−N=2.17 Å distance is the one connecting the upper and lower layers, see Figures 3 a) and 3 d). The third Hf−N=2.52 Å distance is essentially nonbonding. For the isolated carbodiimide unit, see Figure 3 d), there are two C−N=1.24 Å double bonds and a linear N=C=N shape, just as expected. In a sense, this crystal structure is topologically reminiscent of the recently reported bismuth oxide carbodiimide, Bi_2_O_2_(NCN), consisting of layers of [Bi_2_O_2_]^2+^ and [N=C=N]^2−^.^[29]^ In addition, Bi_2_O_2_NCN has been confirmed suitable as a photoanode for photochemical water oxidation, just like the predicted *T′*_2_N_2_(NCN) as will be discussed later.

The second *Cmc*2_1_‐type *T′*_2_CN_4_ candidate, depicted in Figure 3 b), is easily identified as a nitride guanidinate *T′*_2_N(CN_3_) but crystallizes with a lower symmetry than the previously predicted *T*CN_3_ guanidinates, mirrored by the irregular *T′*N_7_ decahedron and the slightly distorted planar CN_3_^5−^ anion, see Figure 3 e). The Hf−N distances in the HfN_7_ decahedron range from 2.09 to 2.34 Å, wider than before. As for the CN_3_^5−^ unit, the C−N bonds arrive at 2×1.35 Å and 1.40 Å, the angles being 115° and 128°, similar to Yb(CN_3_H_4_)_3_.^[18]^

Third, there is the primarily sought *T′*_2_CN_4_ class of phases crystallizing in *P* $\bar{4}$ 2_1_ *c*, given in Figure 3 c), the one that has never been observed before. In that crystal structure, Hf is coordinated by six N with 2×2.11 Å and 4×2.23 Å to form a distorted edge‐ and corner‐sharing HfN_6_ octahedron. The crucial CN_4_^8−^ unit, corresponding to an ortho‐nitrido carbonate (*onc*) anion, contains four identical C−N bonds of 1.49 Å, slightly larger than those of the known carbodiimides and guanidinates. Judged by the N−C−N angles of 106° and 117°, the *onc* unit is almost tetrahedral and conforms to *D*_2*d*_ symmetry, see Figure 3 f). We will further analyze the different chemical behavior to be expected from those differing structures.

### Chemical Bonding and Electronic Structure

**Figure 4:**
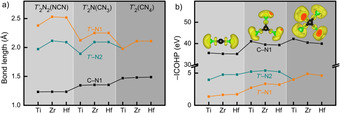
(a) Bond lengths and (b) integrated crystal orbital Hamilton population (ICOHP) of (the sum of) various bonds in *T′*_2_N_2_(NCN), *T′*_2_N(CN_3_), and *T′*_2_CN_4_. For simplicity, we designate C−N1 as the shortest C−N bond whereas *T′*−N1 is the shortest *T′*−N bond. The inset in (b) is the electron localization function (ELF) of anionic groups, with an isosurface level of 0.8.

Because we are mostly interested in the behavior of the complex C/N‐based anions of the *T′*_2_CN_4_ formula, let us focus on them first. For the nearest intraionic C−N bonds (dubbed C−N1) listed in Figure 4 a), the bond lengths slightly increase as we go from nitride carbodiimides *T′*_2_N_2_(NCN) to nitride guanidinates *T′*_2_N(CN_3_) to *onc T′*_2_(CN_4_), a trivial function of the increasing coordination number of the central C atom; a similar course is not found for the *T′*−N distances. That is to say that the C−N1 bond slightly weakens but the larger number of C−N bonds upon going from carbodiimide (2) to guanidinate (3) to *onc* (4) must increase covalency as a whole. On the other side, there is a changing and likewise trivial trend of the *T′*−N distances, consistent with the changing ionic radii. If we take the *onc* structure, for example, in which *T′* is sixfold coordinated, Shannon's ionic radii for such coordination are 0.61 Å for Ti^4+^, 0.72 Å for Zr^4+^, and 0.71 Å for Hf^4+^,^[27]^ and the course runs parallel to what is found theoretically. In fact, the situation is a bit more complex because one finds two types of a *T′*−N bond, the slightly longer *T′*−N1 and slightly shorter *T′*−N2, Figure 3 f). For *T′*_2_N_2_(NCN) and *T′*_2_N(CN_3_), the *T′*−N1 distance is significantly longer than the *T′*−N2 distance, so the existence of the isolated N^3−^ anion in *T′*_2_N_2_(NCN) and *T′*_2_N(CN_3_) is quite obvious even from geometry.

The strengths of the chemical bonds are directly quantified by the Integrated Crystal Orbital Hamilton Populations (ICOHP) as projected by LOBSTER,^[30]^ plotted in Figure 4 b). For *T′*_2_N_2_(NCN) and *T′*_2_N(CN_3_), the covalent part of the *T′*−N bonding is not too large, as expected for a metal‐nitrogen bond. As for the much more covalent and stronger intraionic C−N1 bond, the corresponding strength in the entire complex anions increases from carbodiimide to guanidinate to *onc*, see Figure 4 b), as a function of increasing condensation. This can also be illustrated in color by the socalled electron localization function (ELF)^[31]^ of the N=C=N^2−^, CN_3_^5−^ and CN_4_^8−^ units (for *T′*=Hf), as displayed in Figure 4 b) using an isosurface level of 0.8. In the language of ELF, the clouds around the N atoms indicate “lone‐pair” electrons while the “localized” ones are visible, at least in principle, between C and N. ICOHP directly and numerically quantifies the higher covalency of the CN_4_^8−^ anion.

**Figure 5:**
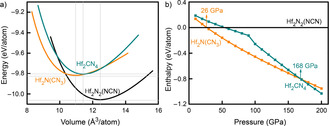
(a) Equation‐of‐state (EOS) fits for the total energies per atom as a function of volume for Hf_2_N_2_(NCN), Hf_2_N(CN_3_), and Hf_2_CN_4_; b) enthalpy‐pressure course of the three predicted compounds.

Questions of relative stability as a function of condensation (or volume) are most easily answered from energy‐volume plots. In order to do so, Vinet equations‐of‐state were calculated for Hf_2_N_2_(NCN), Hf_2_N(CN_3_), and Hf_2_CN_4_ to directly provide that information, see Figure 5 a).^[32]^ It immediately turns out that, under standard conditions, the nitride carbodiimide Hf_2_N_2_(NCN) may be considered the most stable compound (see also convex‐hull discussion below), the chemical ground state, while the nitride guanidinate Hf_2_N(CN_3_) and the ortho‐nitrido carbonate Hf_2_CN_4_ are the metastable ones. As the pressure increases, see Figure 5 b), Hf_2_N_2_(NCN) will transform into Hf_2_N(CN_3_) at about 26 GPa and, at about 168 GPa, into Hf_2_CN_4_. On the other hand, it is puzzling that the spatial requirement of the different anions, as given at zero pressure, does not run parallel to the condensed nature of the complex anions. As seen from Figure 5 a), a nitride guanidinate is more densely packed than the *onc* while *onc* is still better packed than the nitride carbodiimide, so the mutual fit of the Hf−N bonds or the packing itself also must play a role. Given sufficient pressure, however, the nitride carbodiimide will condense into a nitride guanidinate, and a nitride guanidinate will condense into an ortho‐nitrido carbonate, so the highly covalent bonds eventually determine the effective volume.

Before discussing other physical properties, two additional chemical questions must be answered, at least tentatively, as regards absolute thermochemical stability and chemical inertness under laboratory conditions. With respect to the first question, we have calculated possible decomposition pathways and theoretical phase diagrams (Figure S3). Confirming the prior arguments, Hf_2_N_2_(NCN) is the true ground state by −0.24 eV per Hf atom, thermodynamically stable against the convex hull, and should be straightforward to make. Surprisingly enough, Hf(NCN)_2_,^[28]^ previously made by the Meyer group, turns out as being *unstable* by +0.08 eV. Its existence and strong inertness, even against water and air, points towards large activation barriers, a common phenomenon involving complex C/N‐containing anions (see below).

As regards Hf_2_N(CN_3_) and Hf_2_(CN_4_), they are prone to decay by +0.55 and +0.62 eV per Hf atom, whereas TaCN_3_ is unstable by +1.41 eV per Ta atom. That being said, the Ta phase is indeed less likely (but not impossible) whereas the two Hf phases would just need substantial kinetic barriers, larger than in the case of the known Hf(NCN)_2_. While we have been unable to carry out the necessary activation‐barrier calculations, partly due to the sheer complexity (far more complex than, say, the graphite‐diamond problem), partly due to our restricted computational facilities, other semiquantitative arguments are very much in favor of such large kinetic barriers. First, any decomposition of a carbodiimide, guanidinate, or ortho‐nitrido carbonate will involve C−N or C=N covalent bond breaking, on the order of 305–615 kJ mol^−1^ (3.2–6.4 eV),^[33]^ and this is unlikely to begin with; this is also what makes diamond being inert for eternities. Second, electrostatic reasoning points into the same direction, as the LOBSTER‐calculated Madelung energies (per Hf atom) arrive at −31.9 eV for Hf_2_N_2_(NCN), −28.1 eV for Hf_2_N(CN_3_), and −32.0 eV for Hf_2_(CN_4_). Not only must these impressive energies be overcome for decomposition, they go back to the highly charged CN_3_^5−^ and CN_4_^8−^ anions and favor such densely packed high‐pressure phases. As a side note, we reiterate that the (even smaller) Madelung energy of Cr_2_(NCN)_3_ makes this unstable carbodiimide inert even at high temperatures as well as in acidic to alkaline media between pH 1–14.^[14b]^

This brings us to the second question targeting chemical stability which can only be answered experimentally. For example, some carbodimiides such as Hf(NCN)_2_, Cr_2_(NCN)_3_, PbNCN, etc. are perfectly inert in water,[^14b^, ^28^, ^34^] sometimes simply due to surface passivation, others such as Li_2_NCN, Na_2_NCN, or CaNCN are not.[^13a^, ^35^] Hence, we truly need the experiment to corroborate the aforementioned signposts as regards activation barriers and to test the surface stability against a typical laboratory atmosphere.

**Figure 6:**
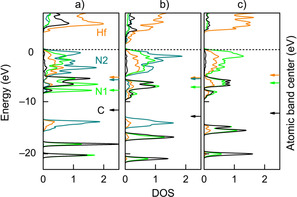
Calculated densities of states (DOS) for a) nitride carbodiimide Hf_2_N_2_(NCN), b) nitride guanidinate Hf_2_N(CN_3_), and c) ortho‐nitrido carbonate Hf_2_CN_4_. Orange, black and green lines represent Hf, C and N, respectively. Arrows on the right axis represent the atomic band centers below the Fermi level.

Coming back to physical properties, the metal‐nonmetal interactions (i.e., covalent part of the Hf−N bonds) mirror what goes on between conduction and valence band, so they determine the band gap, as shown in the calculated densities of states (DOS) for Hf_2_N_2_(NCN), Hf_2_N(CN_3_) and Hf_2_CN_4_, given in Figure 6. Clearly, the nitrogen DOS spreads over the entire energy window through interaction with Hf and C, and the difference between an isolated N^3−^ nitride and a C‐bonded nitrogen is easy to spot. As regards the band centers (shown as arrows on the right), the energetic proximity of Hf and N also indicates some covalent interactions in general, as already seen from COHP analysis, and the DOS shapes clearly broaden upon going from a) to b) to c), so the C−N covalency also strengthens and becomes maximized for the most condensed ortho‐nitrodo carbonate, as expected.

**Figure 7:**
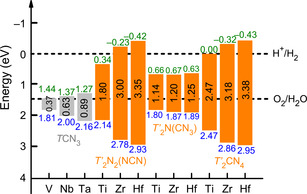
Calculated HSE06 band gaps for stable compounds of the composition *T*CN_3_ (*T*=V, Nb, Ta) and *T′*_2_CN_4_ (*T′*=Ti, Zr, Hf), the latter grouped into nitride carbodiimides, nitride guanidinates, and ortho‐nitrido carbonates. The band‐edge potentials are referenced to the reversible hydrogen electrode. Grey and orange colors indicate the band gap itself whereas green and blue colors represent the conduction band minimum (CBM) and valence band maximum (VBM), respectively.

The hybrid functional HSE06 was chosen to arrive at the most reliable band‐gap values that are depicted in Figure 7.^[36]^ For each composition and crystal structure, the band gap (grey and orange patterns) obviously increases with an increasing atomic number of the metal atom. It is straightforward to correlate this behavior with the course of the lowering Pauling electronegativities (Ti > Zr > Hf, and V> Nb > Ta), so the metal−N interactions become more ionic (larger band gap) upon going down each group of transition metals. What is puzzling, however, is the fact that the band‐gap character generally differs between the different classes of compounds. Clearly, the pure guanidinates *T*CN_3_ show the smallest band gaps, followed by the nitride guanidinates *T′*_2_N(CN_3_), then followed by the nitride carbodiimides *T′*_2_N_2_(NCN) and ortho‐nitrido carbonates *T′*_2_(CN_4_) which are comparable to each other.

### Photochemical Water Splitting

Likewise, the band‐edge potentials were calculated with respect to the reversible hydrogen electrode (RHE), including the conduction band minimum (CBM) and the valence band maximum (VBM) estimated by the semiconductor electronegativity concept (Supporting Information),^[37]^ see again Figure 7. The VBM and CBM of the nitride carbodiimides *T′*_2_N_2_(NCN) and the ortho‐nitrido carbonates *T′*_2_CN_4_ bracket the water redox energy range for *T′*=Zr and Hf, and for these elemental combinations they would achieve water splitting without any external bias voltage (EBV). The CBM of the other materials, however, locate under the H^+^/H_2_ energy level, indicating the necessary EBV for water splitting. On the other hand, the band‐gap value limits the type of light being harvested. As for *T′*=Zr and Hf, *T′*_2_N_2_(NCN) and *T′*_2_CN_4_ are the near ultraviolet (from 305 to 367 nm) light‐harvesting materials. As for the materials with a slightly too narrow band gap, thereby requiring a small EBV, that is, nitride carbodiimide Ti_2_N_2_(NCN) and *onc* Ti_2_CN_4_, they are capable to harvest the red (689 nm) and green light (502 nm), respectively. As such, the nitride carbodiimides *T′*_2_N_2_(NCN) and ortho‐nitrido carbonates *T′*_2_CN_4_ have been identified as potential candidates for water splitting. Especially for the first ones, the layered structures should turn out as particularly useful for making almost 2D‐like crystals, which would provide additional potential as multifunctional compounds.^[38]^

### Nonlinear Optics

Within the ortho‐nitrido carbonates of the *T′*_2_(CN_4_) compounds, the complex CN_4_^8−^ anion itself comprises a large number of valence electrons (32) that may be shifted around and lead to electronic polarization. In combination with the tetrahedral character of this unit and a significant band gap, those *onc* compounds are therefore expected as being good candidates for nonlinear optical (NLO) applications, the NLO data calculated within modern polarization and density‐functional perturbation theory (DFPT) as implemented in ABINIT.^[39]^ As for the visual spectrum from 400 to 700 nm, Ti_2_CN_4_ is predicted to show a second harmonic generation (SHG) coefficient of |*d*_36_|=10.35 pm V^−1^ for the cutoff wavelength of *λ*=502 nm, which is comparable to the known AgGaS_2_ with *d*_36_=13.0 pm V^−1^ for *λ*=454 nm.^[40]^ As for the ultraviolet‐visible (UV/Vis) regime extending from 100 to 400 nm, *T′*_2_CN_4_ with *T′*=Zr and Hf have SHG coefficients of 3.96 and 2.62 pm V^−1^, with *λ*=390 and 367 nm, respectively. This is lower than for the known NLO phase BaGaS_2_ with 12.6 pm V^−1^ for *λ*=346 nm.^[41]^ Nonetheless, *onc*‐type *T′*_2_CN_4_ with the distinct CN_4_^8−^ anion opens yet another NLO option in this field. In addition, the calculated birefringence shown in Table 1 is larger than 0.1, as derived from their refractive index (Figure S4).

**Table 1:** Calculated NLO properties of ortho‐nitrido carbonates *T′*_2_CN_4_

	λ (nm)	SHG coefficients (pm V^−1^)	Δ*n* (nm)
Ti_2_CN_4_	502	*d*_36_=−10.35	0.123
Zr_2_CN_4_	390	*d*_36_=−3.96	0.160
Hf_2_CN_4_	367	*d*_36_=2.62	0.127

### Mechanical Properties

For practical fabrication and device applications, the mechanical properties of the *T′*_2_CN_4_ composition which are computationally accessible by VASPKIT^[42]^ must be studied (Table 2). For being brief, we here focus on the Hf compounds. Despite the sp^3^‐like carbon within ortho‐nitrido carbonate Hf_2_CN_4_, the calculated bulk modulus *K=*212 GPa and Young's modulus *Y*=250 GPa, indicating its resistant ability to compression and stiffness, are much smaller than diamond's sp^3^‐carbon (*K=*435, *Y*=1120 GPa), simply due to the much softer Hf−N bonds. The compound's universal elastic anisotropy *A*_u_=0.30, however, is close to that of diamond (0.27). In addition, the “softness” of the Hf−N interaction differs, as a function of the different chemical functionality, between the three types of compounds. For example, the negative Cauchy pressure or *K*/*G*<1.75 indicate the brittleness of the nitride guanidinate Hf_2_N(CN_3_).^[43]^ The nitride carbodiimide Hf_2_N_2_(NCN) and the ortho‐nitrido carbonate Hf_2_CN_4_, however, are expected to show a more ductile behavior. Finally, the values of the minimum lattice thermal conductivity as derived from the elastic constants indicate the potential of all three compounds in terms of good thermal conductivity.

**Table 2:** Mechanical properties of the Hf_2_CN_4_ compositions adopting different chemical structures. Calculated bulk modulus *K* (GPa), shear modulus *G* (GPa), Young's modulus *Y* (GPa), universal elastic anisotropy index *A*_u_, Cauchy pressure *C* (GPa), brittleness <1.75 < toughness *K*/*G*, minimum lattice thermal conductivity *κ*_min_ (W mK^−1^).

	*K*	*G*	*Y*	*A*_u_	*C*	*K*/*G*	*κ*_min_
Hf_2_N_2_(NCN)	168	40	111	1.7	130	4.2	1.0
Hf_2_N(CN_3_)	182	113	280	0.6	−44	1.6	1.4
Hf_2_(CN_4_)	212	96	250	0.3	37	2.2	1.4

## Conclusion

We have quantum‐mechanically predicted the existence of twelve stable transition‐metal compounds involving complex N‐based anions of dimensionality 1 to 3, including reasonable reaction pathways for their exothermic metathesis as well as high‐pressure approaches. As an obvious starting point, nitride carbodiimides such as Hf_2_N_2_(NCN) turn out as thermochemically stable and should be straightforward to synthesize. The more innovative types of those compositions are given by the first H‐free transition‐metal guanidinates *T*CN_3_ with *T*=V, Nb, Ta, and the ortho‐nitrido carbonates *T′*_2_CN_4_ with *T′*=Ti, Zr, Hf, the latter representing the most condensed and covalent ones of all. While being moderately unstable in terms of the convex hull, both covalent and ionic arguments are in strong favor of substantial kinetic barriers against their decay. In addition, the calculated electronic structures show that all twelve stable phases are semiconductors whose calculated band edges indicate Hf_2_CN_4_ to be the best candidates for photochemical water splitting, the nitride carbodiimides *T′*_2_N_2_(NCN) also showing good potential. Finally, the combined characters of strong C−N bond covalency, large band gap, and acentric symmetry suggests the ortho‐nitrido carbonates *T′*_2_CN_4_ as a novel type of NLO materials, with *T′*=Ti showing the highest SHG coefficient of 10.35 pm V^−1^.

**Supporting Information**. Computational details, calculated phonon band structures, formation energies of the exothermic reactions, convex‐hull discussion, Madelung energies and theoretical phase diagrams, birefringence, structural parameters and elastic constants of twelve compounds.

## Conflict of interest

The authors declare no conflict of interest.

## Supporting information

As a service to our authors and readers, this journal provides supporting information supplied by the authors. Such materials are peer reviewed and may be re‐organized for online delivery, but are not copy‐edited or typeset. Technical support issues arising from supporting information (other than missing files) should be addressed to the authors.

Supplementary
