# Supplementary material for: Predicting Nitrogen‐Based Families of Compounds: Transition‐Metal Guanidinates TCN3 (T=V, Nb, Ta) and Ortho‐Nitrido Carbonates T′ 2CN4 (T′=Ti, Zr, Hf)
Source: Angew Chem Int Ed Engl. 2020 Oct 29;60(1):486–92. doi: 10.1002/anie.202011196 (PMC7821139; doi:10.1002/anie.202011196)
Supplement: Supplementary file 1 — Supplementary [file ANIE-60-486-s001.pdf]

Angewandte Chemie
Eine Zeitschrift der Gesellschaft Deutscher Chemiker

Supporting Information

Predicting Nitrogen-Based Families of Compounds: Transition-Metal Guanidinates $\mathit{T}CN_3$ (T = V, Nb, Ta) and Ortho-Nitrido Carbonates T'_2CN_4 (T' = Ti, Zr, Hf)

*Dongbao Luo, Xianji Qiao, and Richard Dronskowski**

anie_202011196_sm_miscellaneous_information.pdf

Computational details

Computational searches for compounds with the fixed compositions TCN_3 (T = V, Nb, Ta) and T'_2CN_4 (T' = Ti, Zr, Hf) were performed with the CALYPSO structure prediction suite, allowing for up to four formula units per unit cell.[1-2] The Vienna *Ab initio* Simulation Package (VASP) based on the projector augmented wave (PAW) method was used to optimize the structures.[3] The exchange-correlation functional was described by the generalized-gradient approximation (GGA) as envisioned by Perdew-Burke-Ernzerhof (PBE).[4] A plane-wave basis-set cutoff of 900 eV and the Monkhorst–Pack scheme with the ***k***-point density ranging from 23660 to 30240 ***k***-points×atoms were found to converge energies to smaller than 0.1 meV atom^{-1}.[5] Phonon calculations were performed by using a supercell approach with the finite displacement method as done in the Phonopy code.[6] An electron localization function (ELF) was used to visualize the degree of electron localization.[7] The hybrid functional HSE06 was adopted to obtain accurate band gaps.[8] COHP and DOS were projected using the LOBSTER suite which was also utilized to calculate Löwdin charges and Madelung energies, too, directly from the plane waves.[9] The NLO properties were calculated within the framework of modern polarization theory and the density-functional perturbation theory (DFPT) implemented in the ABINIT code.[10] The mechanical properties were calculated by using the VASPKIT tool.[11]

The energy positions of the conduction band (CB) and valence band (VB) edge were estimated by the concept of the semiconductor electronegativity[12]

$$\chi(S) = \sqrt[N]{\chi_1^n \chi_2^s \cdots \chi_{n-1}^p \chi_n^q},$$

$$E_{\mathrm{CB}}^0 = \chi(S) - E^e - \frac{1}{2} E_{\mathrm{g}},$$

where χ_x^y is the electronegativity of the constituent atom with the subscript x denoting the atomic numbering and the superscript y the stoichiometric coefficient;

N is the total number of atoms in the compound;

E_{CB}^0 is the CB edge at the point of zero charge;

E^e is the free-electron energy on the hydrogen scale (ca. 4.5 eV);

E_{g} is the HSE06 band gap.

Supplementary Figures

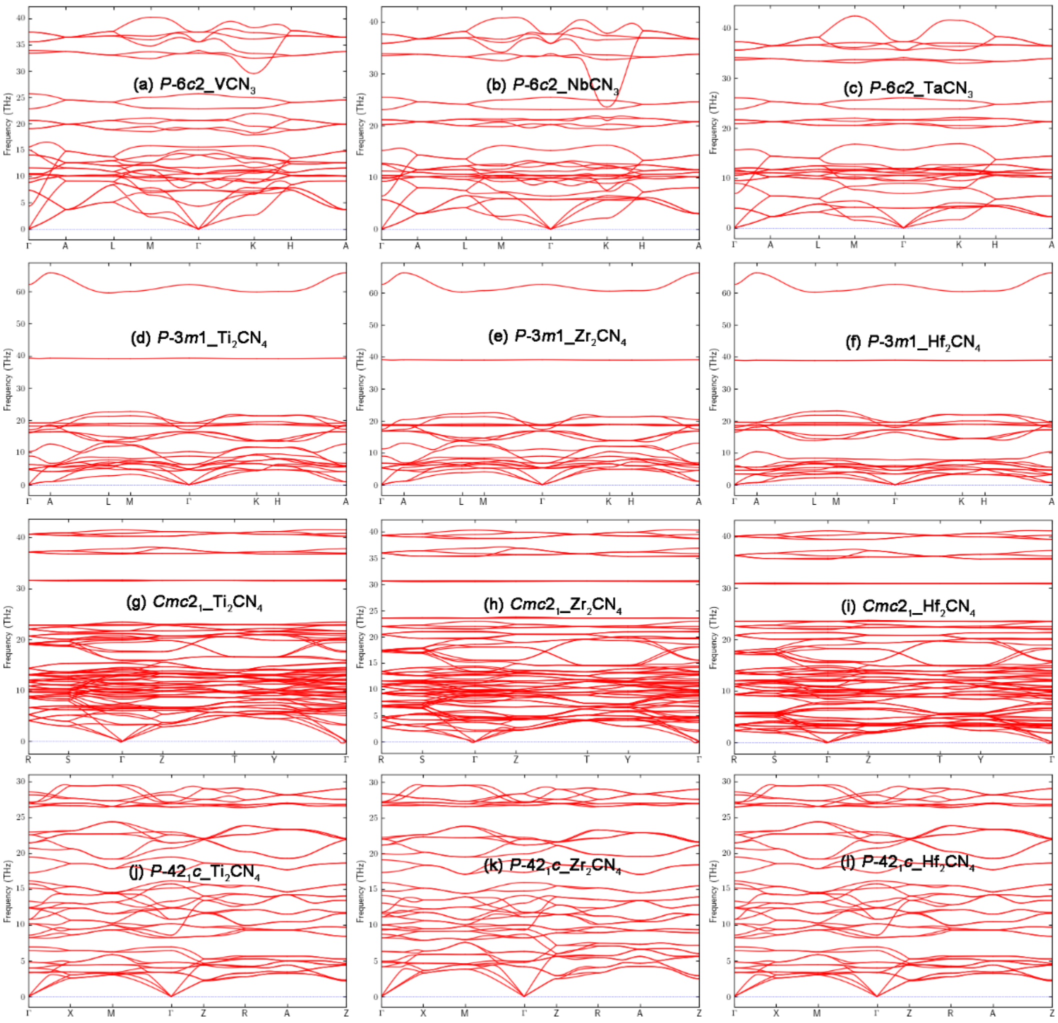

Figure S1. Calculated phonon dispersion curves for compounds of the *T*CN_3 (*T* = V, Nb, Ta) and *T'*$_2CN_4$ (*T'* = Ti, Zr, Hf) compositions.

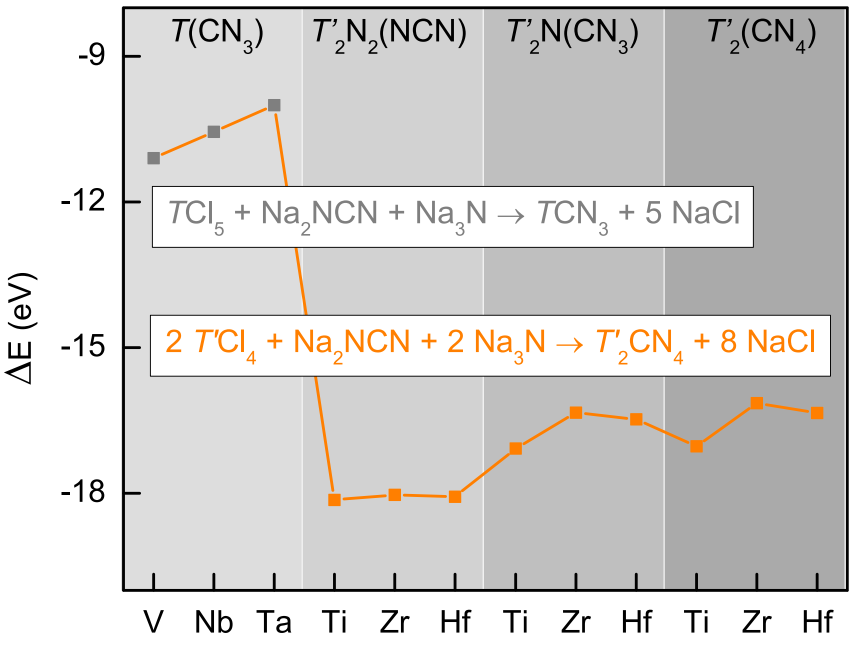

Figure S2. Exothermic reactions for TCN_3 (T = V, Nb, Ta) and T'_2CN_4 (T' = Ti, Zr, Hf) compositions.

To find the most likely decomposition pathways, we constructed the ternary phase diagrams for both Hf–C–N and Ta–C–N systems, including those compositions as targets. Figure S3 (left) evidences the predicted carbodiimide $Hf_2N_2(NCN)$ as thermodynamically stable with respect to path (1). In sharp contrast, the previously synthesized $Hf(NCN)_2$[13] is an unstable compound that should decompose, by path (2), but it clearly does not; $Hf(NCN)_2$ is stable against water and air, so significant activation barriers must exist. Paths (3) and (4) evidence $Hf_2N(CN_3)$ and $Hf_2(CN_4)$ as unstable compounds above the convex hull but their kinetic barriers will be even larger than for $Hf(NCN)_2$. All reaction enthalpies are given per Hf atom:

$$2\ Hf_3N_4 + 3\ C + 2\ N_2 \rightarrow 3\ Hf_2N_2C(NCN) \qquad \Delta H = -0.24\ \text{eV} \qquad (1)$$

$$Hf_2N_2(NCN) + 3\ C + 2\ N_2 \rightarrow 2\ Hf(NCN)_2 \qquad \Delta H = +0.08\ \text{eV} \qquad (2)$$

$$2\ Hf_3N_4 + 3\ C + 2\ N_2 \rightarrow 3\ Hf_2N(CN_3) \qquad \Delta H = +0.55\ \text{eV} \qquad (3)$$

$$2\ Hf_3N_4 + 3\ C + 2\ N_2 \rightarrow 3\ Hf_2(CN_4) \qquad \Delta H = +0.62\ \text{eV} \qquad (4)$$

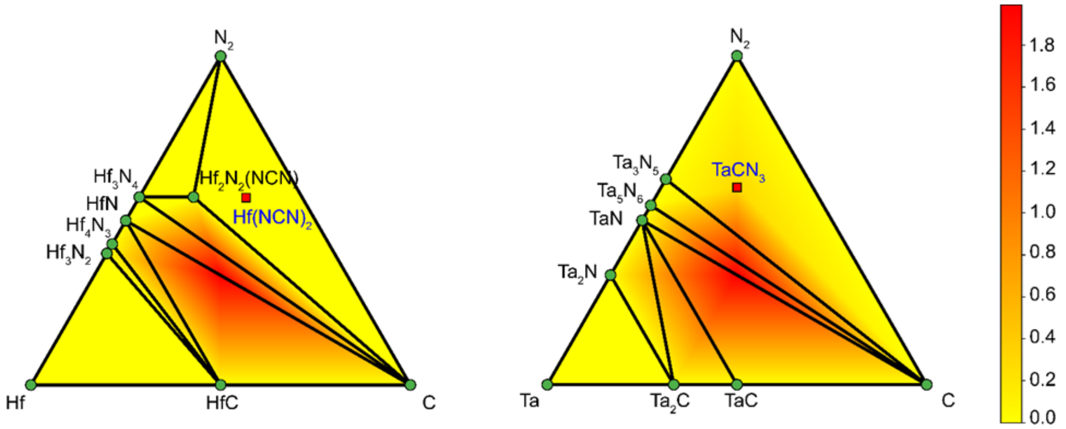

Figure S3. Calculated Hf–C–N and Ta–C–N phase diagrams at ambient pressure, with all referenced phases taken from experiment.[13,16] Green and red circles represent thermodynamically stable and unstable compounds.

When based on Löwdin charges as derived from plane waves (LOBSTER),[9,14] the calculated Madelung energies (per Hf atom) arrive at –31.9 eV for $Hf_2N_2C(NCN)$, at –28.1 eV for $Hf_2N(CN_3)$, and at an impressive –32.0 eV for $Hf_2(CN_4)$ which must be overcome in terms of electrostatics alone. Bond breaking (C–N and C=N) will need an additional amount, on the order of 305–615 kJ mol^{-1} (or 3.2–6.4 eV), for chemical decomposition.[15]

Likewise, Figure S3 (right) shows the predicted guanidinate $TaCN_3$ to be thermodynamically unstable (enthalpy per Ta atom) above the convex hull but we suspect significant activation barriers, just like for the Hf phases.

$$Ta_3N_5 + 3\ C + 2\ N_2 \rightarrow 3\ TaCN_3 \qquad \Delta H = +1.41\ \text{eV}$$

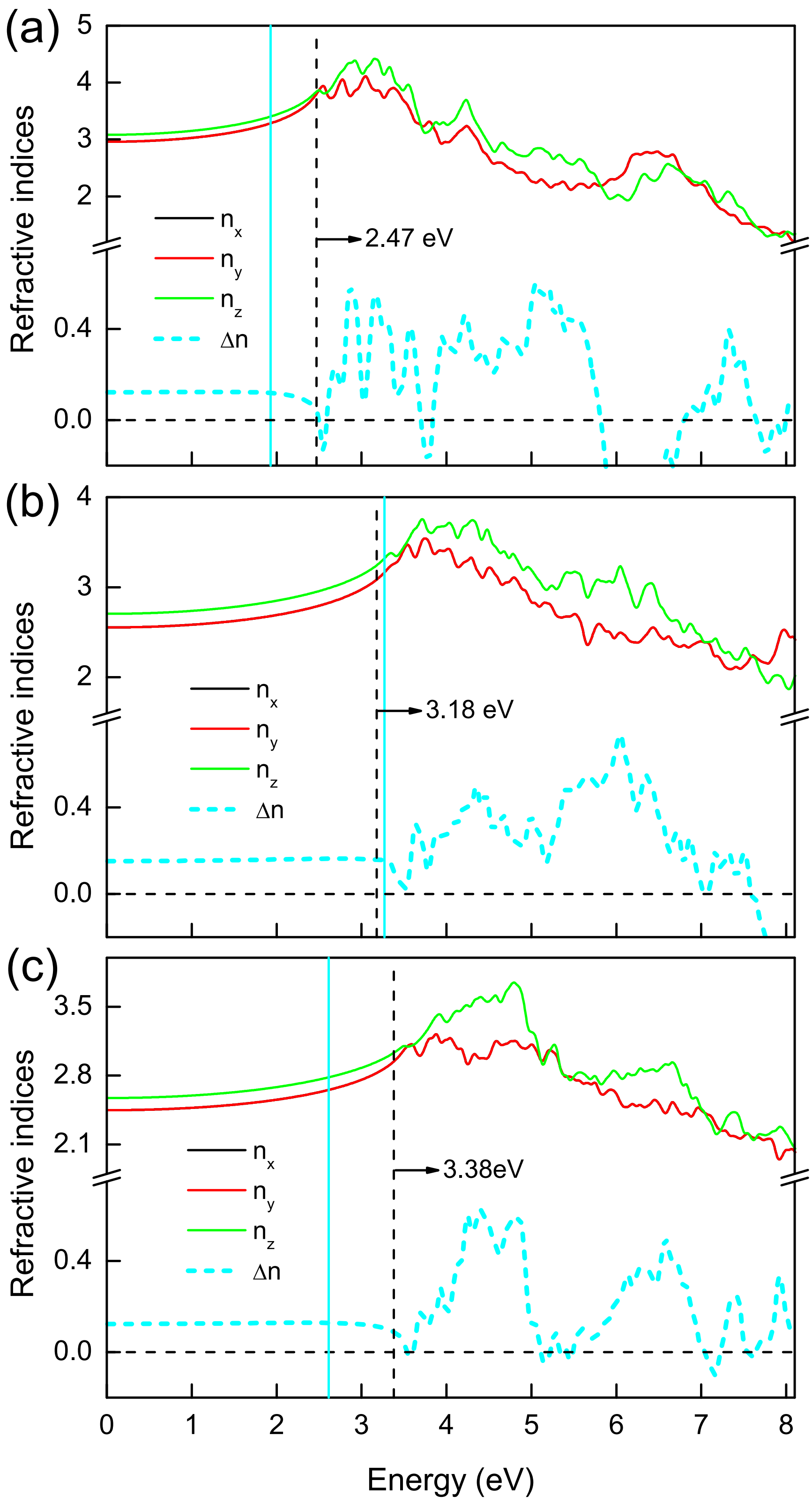

Figure S4. Refractive index for T'_2CN_4 (T' = Ti, Zr, Hf).

Supplementary Tables

Table S1. Detailed structural information of TCN$_3$ (T = V, Nb, Ta) and T'_2CN$_4$ (T' = Ti, Zr, Hf) compositions.

**Phase /Space group**	**Lattice Parameters (Å)**	**Atomic coordinates**
VCN_3<br>$P\bar{6}c2$	a = 4.708<br>b = 4.708<br>c = 5.265<br>α = 90.000<br>β = 90.000<br>γ = 120.000	V<br>⅔ ⅓ 0<br>⅔ ⅓ ½<br>C<br>⅓ ⅔ ¼<br>⅓ ⅔ ¾<br>N<br>0.574 0.983 ¼<br>0.017 0.590 ¼<br>0.410 0.426 ¼<br>0.017 0.426 ¾<br>0.410 0.983 ¾<br>0.574 0.590 ¾
$NbCN_3$<br>$P\bar{6}c2$	a = 4.855<br>b = 4.855<br>c = 5.675<br>α = 90.000<br>β = 90.000<br>γ = 120.000	Nb<br>⅔ ⅓ 0<br>⅔ ⅓ ½<br>C<br>⅓ ⅔ ¼<br>⅓ ⅔ ¾<br>N<br>0.564 0.976 ¼<br>0.024 0.588 ¼<br>0.412 0.436 ¼<br>0.024 0.436 ¾<br>0.412 0.976 ¾<br>0.564 0.588 ¾
$TaCN_3$<br>$P\bar{6}c2$	a = 4.848<br>b = 4.848<br>c = 5.682<br>α = 90.000<br>β = 90.000<br>γ = 120.000	Ta<br>⅔ ⅓ 0<br>⅔ ⅓ ½<br>C<br>⅓ ⅔ ¼<br>⅓ ⅔ ¾<br>N<br>0.565 0.977 ¼<br>0.023 0.589 ¼<br>0.411 0.435 ¼<br>0.023 0.435 ¾

		0.411 0.977 ¾<br>0.565 0.589 ¾
$Ti_2N_2(NCN)$<br>$P\bar{3}m1$	a = 3.326<br>b = 3.326<br>c = 7.826<br>α = 90.000<br>β = 90.000<br>γ = 120.000	Ti<br>⅓ ⅔ 0.661<br>⅔ ⅓ 0.339<br>C<br>0 0 0.999<br>N<br>⅔ ⅓ 0.603<br>0.999 0 0.842<br>0 0.999 0.158<br>⅓ ⅔ 0.396
$Zr_2N_2(NCN)$<br>$P\bar{3}m1$	a = 3.570<br>b = 3.570<br>c = 8.076<br>α = 90.000<br>β = 90.000<br>γ = 120.000	Zr<br>⅓ ⅔ 0.664<br>⅔ ⅓ 0.335<br>C<br>0.999 0 0<br>N<br>⅔ ⅓ 0.605<br>0 0 0.847<br>0.999 0.999 0.152<br>⅓ ⅔ 0.394
$Hf_2N_2(NCN)$<br>$P\bar{3}m1$	a = 3.532<br>b = 3.532<br>c = 8.059<br>α = 90.000<br>β = 90.000<br>γ = 120.000	Hf<br>⅓ ⅔ 0.663<br>⅔ ⅓ 0.336<br>C<br>0 0 0<br>N<br>⅔ ⅓ 0.605<br>0 0 0.846<br>0 0 0.153<br>⅓ ⅔ 0.394
$Ti_2N(CN_3)$<br>$Cmc2_1$	a = 4.151<br>b = 4.151<br>c = 7.518<br>α = 90.000<br>β = 90.000<br>γ = 83.258	Ti<br>0.135 0.396 0.840<br>0.864 0.603 0.340<br>0.396 0.135 0.340<br>0.603 0.864 0.840<br>C<br>0.960 0.039 0.600<br>0.039 0.960 0.100<br>N<br>0.078 0.312 0.548<br>0.687 0.921 0.548<br>0.100 0.899 0.753

		0.921 0.687 0.048<br>0.312 0.078 0.048<br>0.899 0.100 0.253<br>0.405 0.594 0.320<br>0.594 0.405 0.820
$Zr_2N(CN_3)$<br>*Cmc*2$_1$	*a* = 4.512<br>*b* = 4.512<br>*c* = 7.838<br>*α* = 90.000<br>*β* = 90.000<br>*γ* = 83.858	Zr<br>0.131 0.398 0.840<br>0.869 0.602 0.340<br>0.398 0.131 0.340<br>0.602 0.869 0.840<br>C<br>0.960 0.040 0.602<br>0.040 0.960 0.102<br>N<br>0.071 0.292 0.549<br>0.708 0.928 0.549<br>0.091 0.908 0.748<br>0.928 0.708 0.049<br>0.292 0.071 0.049<br>0.908 0.091 0.248<br>0.414 0.586 0.324<br>0.586 0.414 0.824
$Hf_2N(CN_3)$<br>*Cmc*2$_1$	*a* = 4.439<br>*b* = 4.439<br>*c* = 7.814<br>*α* = 90.000<br>*β* = 90.000<br>*γ* = 83.830	Hf<br>⅛ 0.398 0.840<br>⅞ 0.602 0.340<br>0.398 ⅛ 0.340<br>0.602 ⅞ 0.840<br>C<br>0.963 0.036 0.601<br>0.036 0.963 0.101<br>N<br>0.076 0.293 0.549<br>0.707 0.924 0.549<br>0.096 0.903 0.749<br>0.924 0.707 0.049<br>0.293 0.076 0.049<br>0.903 0.096 0.249<br>0.405 0.595 0.325<br>0.595 0.405 0.825
$Ti_2(CN_4)$<br>$P\bar{4}2_1c$	*a* = 4.821<br>*b* = 4.821<br>*c* = 5.978<br>*α* = 90.000<br>*β* = 90.000	Ti<br>½ 0 0.357<br>0 ½ 0.643<br>0 ½ 0.143<br>½ 0 0.857

	γ = 90.000	C<br>0 0 ½<br>½ ½ 0<br>N<br>0.121 0.768 0.374<br>0.879 0.232 0.374<br>0.768 0.879 0.626<br>0.232 0.121 0.626<br>0.621 0.732 0.126<br>0.379 0.268 0.126<br>0.268 0.621 0.874<br>0.732 0.379 0.874
$Zr_2(CN_4)$<br>$P\bar{4}2_1c$	a = 5.021<br>b = 5.021<br>c = 6.526<br>α = 90.000<br>β = 90.000<br>γ = 90.000	Zr<br>½ 0 0.352<br>0 ½ 0.648<br>0 ½ 0.148<br>½ 0 0.852<br>C<br>0 0 ½<br>½ ½ 0<br>N<br>0.105 0.774 0.379<br>0.895 0.226 0.379<br>0.774 0.895 0.621<br>0.226 0.105 0.621<br>0.605 0.726 0.121<br>0.395 0.274 0.121<br>0.274 0.605 0.879<br>0.726 0.395 0.879
$Hf_2(CN_4)$<br>$P\bar{4}2_1c$	a = 4.979<br>b = 4.979<br>c = 6.448<br>α = 90.000<br>β = 90.000<br>γ = 90.000	Hf<br>½ 0 0.356<br>0 ½ 0.644<br>0 ½ 0.144<br>½ 0 0.856<br>C<br>0 0 ½<br>½ ½ 0<br>N<br>0.114 0.773 0.379<br>0.886 0.227 0.379<br>0.773 0.886 0.621<br>0.227 0.114 0.621<br>0.614 0.727 0.121<br>0.386 0.273 0.121

		0.273 0.614 0.879<br>0.727 0.386 0.879

Table S2. Elastic constants of TCN$_3$ (T = V, Nb, Ta) and T'_2CN$_4$ (T' = Ti, Zr, Hf) compositions.

**Phase/Space group**	**Elastic tensor C_{ij} (GPa) /Elastic stability criteria**
VCN$_3$<br>$P\bar{6}c2$	$\begin{pmatrix} 288.0 & 106.9 & 37.6 & & & \\ 106.9 & 288.0 & 37.6 & & 0 & \\ 37.6 & 37.6 & 403.3 & & & \\ & & & 90.6 & 0 & 0 \\ & 0 & & 0 & 30.2 & 0 \\ & & & 0 & 0 & 30.2 \end{pmatrix}$<br>Mechanical stability:<br>$C_{11} > \lvert C_{12} \rvert$<br>$2 \cdot C_{13}^2 < C_{33} \cdot (C_{11}+C_{12})$<br>$C_{44} > 0$
NbCN$_3$<br>$P\bar{6}c2$	$\begin{pmatrix} 269.7 & 88.9 & 64.0 & & & \\ 88.9 & 269.7 & 37.6 & & 0 & \\ 64.0 & 64.0 & 422.8 & & & \\ & & & 90.4 & 0 & 0 \\ & 0 & & 0 & 27.5 & 0 \\ & & & 0 & 0 & 27.5 \end{pmatrix}$<br>Mechanical stability:<br>$C_{11} > \lvert C_{12} \rvert$<br>$2 \cdot C_{13}^2 < C_{33} \cdot (C_{11}+C_{12})$<br>$C_{44} > 0$
TaCN$_3$<br>$P\bar{6}c2$	$\begin{pmatrix} 295.1 & 98.2 & 57.3 & & & \\ 98.2 & 295.1 & 57.3 & & 0 & \\ 57.3 & 57.3 & 453.4 & & & \\ & & & 98.4 & 0 & 0 \\ & 0 & & 0 & 26.7 & 0 \\ & & & 0 & 0 & 26.7 \end{pmatrix}$<br>Mechanical stability:<br>$C_{11} > \lvert C_{12} \rvert$<br>$2 \cdot C_{13}^2 < C_{33} \cdot (C_{11}+C_{12})$<br>$C_{44} > 0$
Ti$_2$N$_2$(NCN)<br>$P\bar{3}m1$	$\begin{pmatrix} 197.8 & 154.6 & 124.0 & & & \\ 154.6 & 197.8 & 124.0 & & 0 & \\ 124.0 & 124.0 & 347.4 & & & \\ & & & 21.6 & 0 & 0 \\ & 0 & & 0 & 19.2 & 0 \\ & & & 0 & 0 & 19.2 \end{pmatrix}$

	Mechanical stability:<br>$C_{11} > C_{12}$<br>$C_{13}^2 < 0.5 \cdot C_{33} \cdot (C_{11}+C_{12})$<br>$C_{14}^2 < 0.5 \cdot C_{44} \cdot (C_{11}-C_{12})$<br>$C_{44} > 0$
$Zr_2N_2(NCN)$<br>$P\bar{3}m1$	$\begin{pmatrix} 213.8 & 138.8 & 108.5 & & & \\ 138.8 & 213.8 & 108.5 & & 0 & \\ 108.5 & 108.5 & 299.0 & & & \\ & & & 37.5 & 0 & 0 \\ & 0 & & 0 & 21.2 & 0 \\ & & & 0 & 0 & 21.2 \end{pmatrix}$<br>Mechanical stability:<br>$C_{11} > C_{12}$<br>$C_{13}^2 < 0.5 \cdot C_{33} \cdot (C_{11}+C_{12})$<br>$C_{14}^2 < 0.5 \cdot C_{44} \cdot (C_{11}-C_{12})$<br>$C_{44} > 0$
$Hf_2N_2(NCN)$<br>$P\bar{3}m1$	$\begin{pmatrix} 217.8 & 159.1 & 109.2 & & & \\ 159.1 & 217.8 & 109.2 & & 0 & \\ 109.2 & 109.2 & 319.2 & & & \\ & & & 29.3 & 0 & 0 \\ & 0 & & 0 & 22.7 & 0 \\ & & & 0 & 0 & 22.7 \end{pmatrix}$<br>Mechanical stability:<br>$C_{11} > C_{12}$<br>$C_{13}^2 < 0.5 \cdot C_{33} \cdot (C_{11}+C_{12})$<br>$C_{14}^2 < 0.5 \cdot C_{44} \cdot (C_{11}-C_{12})$<br>$C_{44} > 0$
$Ti_2N(CN_3)$<br>$Cmc2_1$	$\begin{pmatrix} 312.9 & 25.9 & 159.1 & & & \\ 25.9 & 449.2 & 105.7 & & 0 & \\ 159.1 & 105.7 & 324.5 & & & \\ & & & 96.6 & 0 & 0 \\ & 0 & & 0 & 154.3 & 0 \\ & & & 0 & 0 & 98.9 \end{pmatrix}$<br>Mechanical stability:<br>$C_{11} > 0$<br>$C_{11} \cdot C_{22} > C_{12}^2$<br>$C_{11} \cdot C_{22} \cdot C_{33}+2 \cdot C_{12} \cdot C_{13} \cdot C_{23} > C_{11} \cdot C_{23}^2+C_{22} \cdot C_{13}^2+C_{33} \cdot C_{12}^2$<br>$C_{44} > 0$<br>$C_{55} > 0$<br>$C_{66} > 0$

$Zr_2N(CN_3)$<br>$Cmc2_1$	$\begin{pmatrix} 305.2 & 20.0 & 124.1 & & & \\ 20.0 & 371.2 & 93.3 & & 0 & \\ 124.1 & 93.3 & 273.6 & & & \\ & & & 73.3 & 0 & 0 \\ & 0 & & 0 & 112.0 & 0 \\ & & & 0 & 0 & 48.6 \end{pmatrix}$<br>Mechanical stability:<br>$C_{11} > 0$<br>$C_{11} \cdot C_{22} > C_{12}^2$<br>$C_{11} \cdot C_{22} \cdot C_{33} + 2 \cdot C_{12} \cdot C_{13} \cdot C_{23} > C_{11} \cdot C_{23}^2 + C_{22} \cdot C_{13}^2 + C_{33} \cdot C_{12}^2$<br>$C_{44} > 0$<br>$C_{55} > 0$<br>$C_{66} > 0$
$Hf_2N(CN_3)$<br>$Cmc2_1$	$\begin{pmatrix} 340.3 & 39.9 & 134.7 & & & \\ 39.9 & 452.3 & 98.2 & & 0 & \\ 134.7 & 98.2 & 298.3 & & & \\ & & & 83.4 & 0 & 0 \\ & 0 & & 0 & 129.3 & 0 \\ & & & 0 & 0 & 77.6 \end{pmatrix}$<br>Mechanical stability:<br>$C_{11} > 0$<br>$C_{11} \cdot C_{22} > C_{12}^2$<br>$C_{11} \cdot C_{22} \cdot C_{33} + 2 \cdot C_{12} \cdot C_{13} \cdot C_{23} > C_{11} \cdot C_{23}^2 + C_{22} \cdot C_{13}^2 + C_{33} \cdot C_{12}^2$<br>$C_{44} > 0$<br>$C_{55} > 0$<br>$C_{66} > 0$
$Ti_2(CN_4)$<br>$P\bar{4}2_1c$	$\begin{pmatrix} 388.4 & 97.7 & 117.2 & & & \\ 97.7 & 388.4 & 117.2 & & 0 & \\ 117.2 & 117.2 & 351.4 & & & \\ & & & 85.3 & 0 & 0 \\ & 0 & & 0 & 79.2 & 0 \\ & & & 0 & 0 & 79.2 \end{pmatrix}$<br>Mechanical stability:<br>$C_{11} > \lvert C_{12} \rvert$<br>$2 \cdot C_{13}^2 > C_{33} \cdot (C_{11} + C_{12})$<br>$C_{44} > 0$
$Zr_2(CN_4)$<br>$P\bar{4}2_1c$	$\begin{pmatrix} 338.0 & 110.4 & 125.8 & & & \\ 110.4 & 338.0 & 125.8 & & 0 & \\ 125.8 & 125.8 & 351.4 & & & \\ & & & 75.9 & 0 & 0 \\ & 0 & & 0 & 59.6 & 0 \\ & & & 0 & 0 & 59.6 \end{pmatrix}$<br>Mechanical stability:<br>$C_{11} > \lvert C_{12} \rvert$

	$2 \cdot C_{13}^2 > C_{33} \cdot (C_{11}+C_{12})$<br>$C_{44} > 0$
$Hf_2(CN_4)$<br>$P\bar{4}2_1c$	$\begin{pmatrix} 377.9 & 126.0 & 133.8 & & & \\ 126.0 & 377.9 & 133.8 & & 0 & \\ 133.8 & 133.8 & 372.1 & & & \\ & & & 89.4 & 0 & 0 \\ & 0 & & 0 & 72.9 & 0 \\ & & & 0 & 0 & 72.9 \end{pmatrix}$<br>Mechanical stability:<br>$C_{11} > \|C_{12}\|$<br>$2 \cdot C_{13}^2 > C_{33} \cdot (C_{11}+C_{12})$<br>$C_{44} > 0$

References

1. Y. C. Wang, J. Lv, L. Zhu, Y. M. Ma, *Phys. Rev. B* **2010**, *82*, 094116.
2. Y. C. Wang, J. Lv, L. Zhu, Y. M. Ma, *Comput. Phys. Commun.* **2012**, *183*, 2063–2070.
3. a) G. Kresse, J. Furthmüller, *Phys. Rev. B* **1996**, *54*, 1169; b) P. E. Blöchl, *Phys. Rev. B* **1994**, *40*, 17953; c) G. Kresse, D. Joubert, *Phys. Rev. B* **1999**, *59*, 1758–1775.
4. J. P. Perdew, K. Burke, M. Ernzerhof, *Phys. Rev. Lett.* **1996**, *77*, 3865–3868.
5. H. J. Monkhorst, J. D. Pack, *Phys. Rev. B* **1976**, *13*, 5188.
6. A. Togo, I. Tanaka, *Scr. Mater.* **2015**, *108*, 1–5.
7. A. D. Becke, K. E. Edgecombe, *J. Chem. Phys.* **1990**, *92*, 5397–5403.
8. J. Heyd, G. E. Scuseria, M. Ernzerhof, *J. Chem. Phys.* **2003**, *118*, 8207–8215.
9. a) R. Dronskowski, P. E. Blöchl, *J. Phys. Chem.* **1993**, *97*, 8617–8624; b) V. L. Deringer, A. L. Tchougréeff, R. Dronskowski, *J. Phys. Chem. A* **2011**, *115*, 5461–5466; c) S. Maintz, V. L. Deringer, A. L. Tchougréeff, R. Dronskowski, *J. Comput. Chem.* **2013**, *34*, 2557–2567; d) S. Maintz, V. L. Deringer, A. L. Tchougréeff, R. Dronskowski, *J. Comput. Chem.* **2016**, *37*, 1030–1035; e) R. Nelson, C. Ertural, J. George, V. L. Deringer, G. Hautier, R. Dronskowski, *J. Comput. Chem.* **2020**, *41*, 1–10.
10. a) X. Gonze, J.-M. Beuken, R. Caracas, F. Detraux, M. Fuchs, G.-M. Rignanese, L. Sindic, M. Verstraete, G. Zerah, F. Jollet, M. Torrent, A. Roy, M. Mikami, Ph. Ghosez, J.-Y. Raty, D.C. Allan, *Comput. Mater. Sci.* **2002**, *25*, 478–492; b) D. R. Hamann, X. F. Wu, K. M. Rabe, D. Vanderbilt, *Phys. Rev. B* **2005**, *71*, 035117.

11. V. Wang, N. Xu, J.-C. Liu, G. Tang, W. T. Geng, *VASPKIT: A Pre- and Post-Processing Program for the VASP Code*, http://vaspkit.sourceforge.net.
12. Y. Xu, M. A.A. Schoonen, *Am. Mineral.* **2000**, *85*, 543–556.
13. K. Dolabdjian, A. Kobald, C. P. Romao, H.-J. Meyer, *Dalton Trans*. **2018**, *47*, 10249–10255.
14. C. Ertural, S. Steinberg, R. Dronskowski, *RSC Adv.* **2019**, *9*, 29821–29830.
15. J. Emsley, *The Elements*, Clarendon Press, Oxford 1991.
16. a) R. B. Russell, *J. Appl. Phys.* **1953**, *24*, 232–233; b) D. E. Nixon, G. S. Parry, A. R. J. P. Ubbelohde, *Proc. Math. Phys. Eng. Sci.* **1966**, *291*, 324–339; c) J. Donohue, *Acta Crystallogr.* **1961**, *14*, 1000–1001; d) P. G. Cotter, J. A. Kohn, *J. Am. Ceram. Soc.* **1954**, *37*, 415–420; e) W. Lengauer, D. Rafaja, R. Täubler, C. Kral, P. Ettmayer, *Acta Metall. Mater.* **1993**, *41*, 3505–3514; f) J. Zhang; A. R. Oganov, X. Li, H. Niu, *Phys. Rev. B* **2017**, *95*, 020103; g) Y. G. Zainulin, S. I. Alyamovskii, G. P. Shveikin, P. V. Geld, *Teplofiz. Vys. Temp.* **1971**, *9*, 546–549; h) A. Zerr, G. Miehe, R. Riedel, *Nat. Mater.* **2003**, *2*, 185–189; i) A. W. Hull, *Science* **1920**, *52*, 227–229; j) F. Lissner, Th. Schleid, *Z. Kristallogr. NCS* **2001**, *216*, 351–352; k) K. Becker, F. Ebert, *Z. Phys.* **1925**, *31*, 268–272; l) L. Liu, K. Huang, J. Hou, H. Zhu, *Mater. Res. Bull.* **2012**, *47*, 1630–1635; m) A. N. Christensen, B. Lebech, *Acta Crystallogr. Sect. B* **1978**, *34*, 261–263; n) A. Fontbonne, J. C. Gilles, *Rev. Int. Hautes Temp. Réfract.* **1969**, *6*, 181–192; o) J. Strähle, *Z. Anorg. Allg. Chem.* **1973**, *402*, 47–57.
